# Establishment and evaluation of an improved rat model of open abdomen

**DOI:** 10.1002/ame2.12376

**Published:** 2023-12-29

**Authors:** Ye Liu, Sicheng Li, Jinjian Huang, Ze Li, Kang Chen, Guiwen Qu, Xiuwen Wu, Jianan Ren

**Affiliations:** ^1^ School of Medicine, Southeast University Nanjing China; ^2^ Research Institute of General Surgery Affiliated Jinling Hospital, Medical School of Nanjing University Nanjing China

**Keywords:** animal models, open abdomen, patch, polypropylene mesh

## Abstract

**Introduction:**

This study aimed to establish an animal model of open abdomen (OA) through temporary abdominal closure via different techniques.

**Methods:**

Adult male Sprague–Dawley rats were randomly divided into three groups: group A (OA with polypropylene mesh alone); group B (OA with polypropylene mesh combined with a patch); and group C (OA with polypropylene mesh and a sutured patch). Vital signs, pathophysiological changes, and survival rates were closely monitored in the rats for 7 days after surgery. Abdominal X‐rays and histopathological examinations were performed to assess abdominal organ changes and wound healing.

**Results:**

The results showed no significant difference in mortality rates among the three groups (*p* > 0.05). However, rats in group B exhibited superior overall condition, cleaner wounds, and a higher rate of wound healing compared to the other groups (*p* < 0.05). Abdominal X‐rays indicated that varying degrees of distal intestinal obstruction in all groups. Histopathological examinations revealed fibrous hyperplasia, inflammatory cell infiltration, neovascularization, and collagen deposition in all groups. Group B demonstrated enhanced granulation tissue generation, neovascularization, and collagen deposition compared to the other groups (*p* < 0.05).

**Conclusions:**

Polypropylene mesh combined with patches is the most suitable method for establishing an animal model of OA. This model successfully replicated the pathological and physiological changes in postoperative patients with OA, specifically the progress of abdominal skin wound healing. It provides a practical and reliable animal model for OA research.

## Graphical abstract

An animal model of open abdomen (OA) was established using different techniques for temporary abdominal closure in rats. The combination of polypropylene mesh and patches resulted in superior wound healing and overall condition compared to other methods. Abdominal X‐rays showed varying degrees of distal intestinal obstruction in all groups, while histopathological examinations revealed common pathological changes in all groups. The developed animal model successfully replicates the physiological and pathological changes observed in postoperative patients with OA, offering a reliable research tool for studying OA.

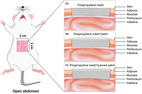

## 1 INTRODUCTION

Open abdomen (OA) refers to leaving the abdominal wall incision open after surgery without sutures to achieve debridement and drainage and to reduce intra‐abdominal pressure.^1^, ^2^, ^3^ OA is a cutting‐edge treatment for severe abdominal combat trauma, infection, and abdominal hypertension.^4^ Usually, temporary abdominal closure (TAC) is required to close the abdominal cavity after opening to avoid unrestricted opening of this cavity.^5^ However, direct contact between the TAC material and the intestinal canal can lead to the occurrence of enteroatmospheric fistula (EAF), which severely limits the application of OA therapy.^6^ Therefore, establishing an effective animal model of OA is essential for studying open abdominal wounds, the pathophysiological mechanisms of EAF occurrence, and developing open abdominal wound protection techniques.

OA models are widely used in biomedical research to study various aspects of abdominal surgery, such as wound healing, infection, adhesion formation, and organ function. However, previously reported animal models have some limitations and shortcomings, such as high animal mortality, low reproducibility, and lack of standardization in modeling methods and assessment approaches, which may affect their validity and applicability in simulating human clinical scenarios.^7^, ^8^, ^9^ The present study aimed to establish a highly clinically relevant, simple, repetitive, low morbidity and mortality OA model by modifying the method previously reported.

## 2 METHODS

### 2.1 Animals

**FIGURE 1:**
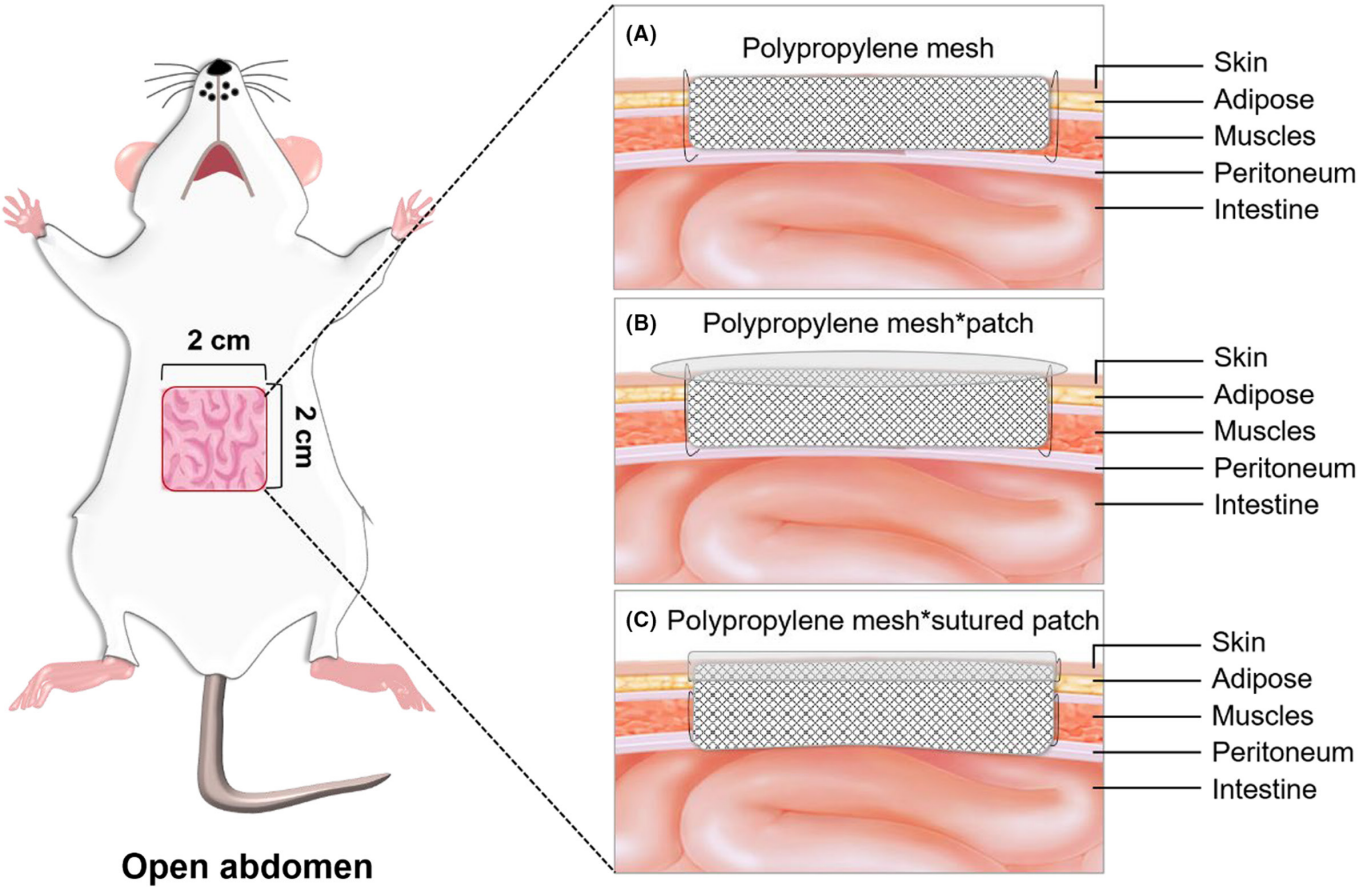
Schematic diagram of the three methods used to construct the OA model. Group A: temporary closure of the abdomen with polypropylene mesh alone; Group B: temporary closure of the abdomen with polypropylene mesh and a patch; Group C: temporary closure of the abdomen with polypropylene mesh and a sutured patch.

Eighteen SPF‐grade male Sprague–Dawley (SD) rats, aged 10–15 weeks, weighing 210–250 g, were provided by Jiangsu Huachuang Xinnuo Pharmaceutical Technology Co. Ltd, TaiZhou, China. The rats were housed in a climate‐controlled environment (22 ± 2°C, 50 ± 10% relative humidity) with 12‐h/12‐h light/dark cycles, one rat per cage. They were supplied with adequate raw chow and water for 2 weeks, then fasted for 12 h before surgery, and drank water freely. As shown in Figure 1, the rats were divided into the following three groups according to the random number table method: group A, temporary closure of the abdomen with polypropylene mesh alone (*n* = 6); group B, temporary closure of the abdomen with polypropylene mesh combined with a patch (*n* = 6); group C, temporary closure of the abdomen with polypropylene mesh and a sutured patch (*n* = 6). The animal disposal and research protocols were approved by the Experimental Animal Ethics Committee of Nanjing Jinling Hospital, and the ethical approval document batch number was 2021DZDWLS‐001. All experiments were performed in compliance with the *Guide for the Care and Use of Laboratory Animals*. The polypropylene mesh (Type: N01, Size: 150 × 150 mm) was purchased from Hebei Rino Medical Equipment Co. Ltd; sodium penicillin for injection was from Shanghai Yuanye Biotechnology Co.

### 2.2 Methods

#### 2.2.1 Anesthesia and preoperative preparation

Rats were anesthetized by inhaling isoflurane until they were confirmed unconscious. The rats were then fixed on the operating table, the skin was prepared, a square incision mark of 2 × 2 cm was made in the middle of the abdomen, the area was disinfected with iodophor, and towels were placed over the area.

#### 2.2.2 Preparation of full abdominal wall defects

The skin was incised along the marker, and the abdominal wall was excised after entering the abdomen layer by layer to prepare a 2 × 2 cm abdominal wall defect, and the field was thoroughly stanched. A heated blanket was used to maintain the body temperature of the model animal at 36–37°C.

#### 2.2.3 Suture fixation of the mesh

Rats with abdominal wall defects were treated as follows. Group A: to prevent the unrestricted opening of the abdominal cavity and retraction of the abdominal wall and to achieve temporary closure of the abdomen, a layer of polypropylene mesh matching the size of the defect was sutured to the edge of the abdominal wall defect with simple interrupted sutures using 4–0 silk thread with a margin of 1.0–1.5 mm and a stitch spacing of 3.0–4.0 mm. The area was then re‐sterilized with iodophor. Group B: the polypropylene mesh was uniformly sutured to the edge of the abdominal wall defect using the above‐mentioned suturing method. The area was then re‐sterilized with iodophor. A 6 × 6 cm medical wound waterproof patch was then applied to the abdominal defect, and the patch was closely adhered to the abdominal skin. Group C: using the suture method described above, the polypropylene mesh was uniformly sutured to the muscle layer of the abdominal wall with 4–0 silk thread, and then the medical patch was sutured to the skin at the edge of the abdominal wall defect with 4–0 silk thread (lateral spacing of 1.5–2.0 mm, stitch spacing of 3.0–4.0 mm). The area was then re‐sterilized with iodophor. The rats in each group were returned to the cage for awakening, given heat insulation, and kept in an SPF‐grade animal house.

#### 2.2.4 Postoperative treatment

After surgery, the rats remained in individual cages under standard conditions. Penicillin was injected intramuscularly for the first 3 days. After 7 days, all rats were euthanized following institutional approval, and detailed evaluation of the main indices described below was performed by the investigators.

### 2.3 Main indices

We set the success criteria for modeling as maintaining survival for 7 days postoperatively and compared the following main indices over 7 days after surgery: (1) General condition of rats: survival rate, vital signs, mental status, diet, defecation, and activity were recorded. (2) Gross observation: wound healing, incisional infection, intestinal abrasion, intestinal adhesions, intestinal fistula were monitored, and the KATADA adhesion score was recorded.^10^ (3) An abdominal X‐ray was performed on postoperative day (POD) 6 to observe abdominal organs. (4) The surrounding tissues of abdominal wall defects were obtained on POD 7, fixed in 10% neutral formalin solution overnight, and processed with Hematoxylin–Eosin (HE) and Masson staining. The physiopathological changes in the tissues were recorded, and histological scores were determined.

### 2.4 Statistical analysis

SPSS 22.0 statistical software was utilized for analysis. The measurement data conforming to the normal distribution were expressed as means ± standard deviation, and a one‐way ANOVA test was adopted first for comparison between groups, followed by the Dunnet *t* test for two‐way comparison; the count data were expressed as rates, and the *χ* ^2^ test or Fisher's exact test was employed for comparison between groups. Differences were statistically significant at *p* < 0.05.

## 3 RESULTS

### 3.1 Survival and general condition of the rats

**FIGURE 2:**
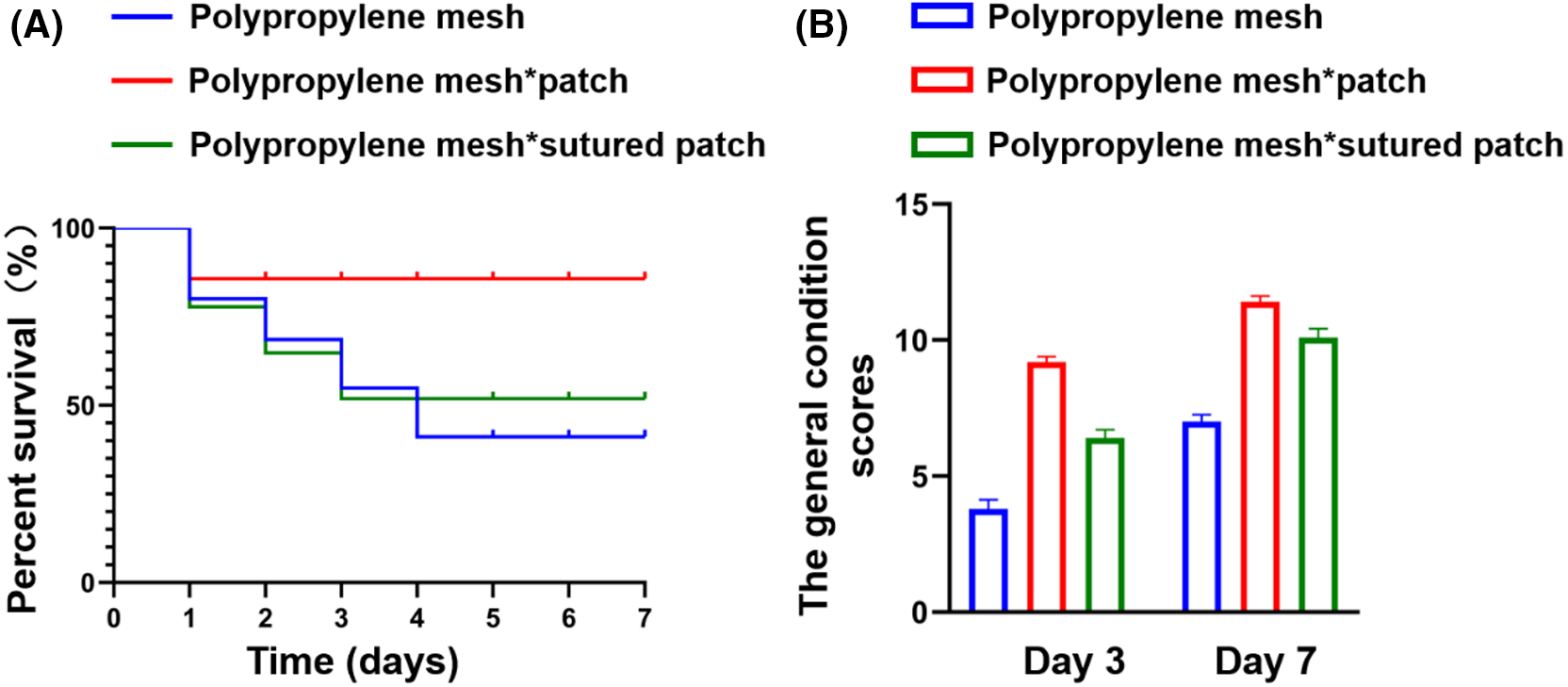
General observations of rats after OA. (A) The percentage survival of rats during the 7 days of the experiment. (B) The general condition scores of rats on experimental days 3 and 7.

We established the OA model using three different methods (Figure 1). During the 7‐day observation period, there was no statistically significant difference in terms of the mortality rate among the three groups (Figure 2A). In addition, we assessed the general postoperative condition of the rats (Table S1). During the first 2 days, all groups exhibited poor mental condition, decreased appetite, slightly reduced fecal output, and significantly diminished activity levels. However, gradual recovery was observed thereafter, with group B demonstrating better overall postoperative recovery compared to the other two groups. As shown in Figure 2B, based on our assessment of general rat conditions, group B displayed significantly higher scores than the other two groups on day 3 and day 7 (*p* < 0.05).

### 3.2 Gross view of the open abdominal wounds

**FIGURE 3:**
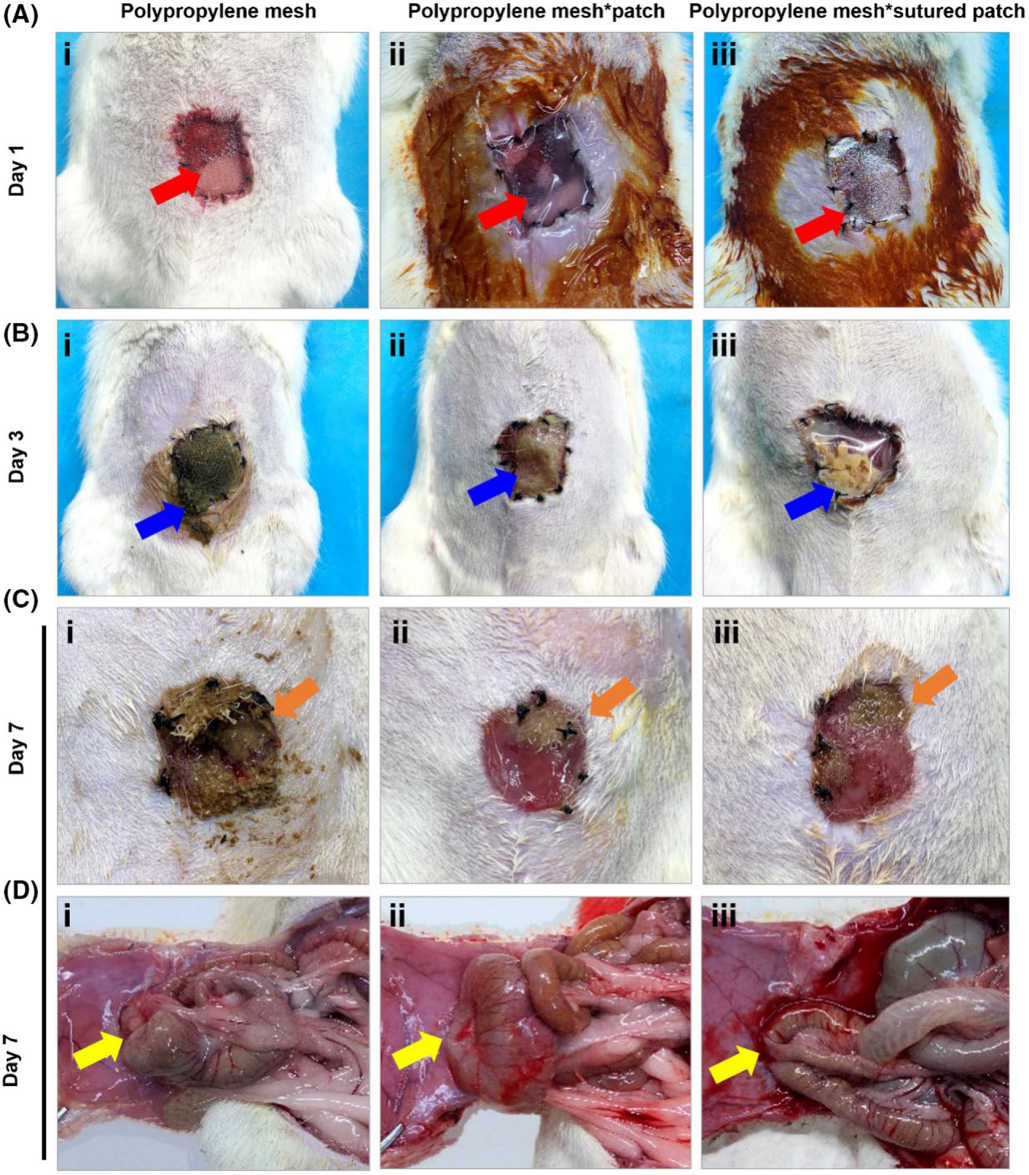
General observations after OA. (A) The open abdominal cavity on day 1 of the different modeling methods; red arrows show the site of abdominal wall defect. (B) The open abdominal cavity on day 3 of the different modeling methods; blue arrows show the polypropylene mesh. (C) The open abdominal cavity on day 7 of the different modeling methods; orange arrows show the regenerative tissue in the abdominal wall defect. (D) The abdominal adhesions on day 7 of the different modeling methods; yellow arrows show the adhesions between the abdominal contents and the abdominal wall.

The abdomen in the three groups on the day of modeling is shown in Figure 3Ai–iii. On POD 3, the group A rats exhibited spillage of intestinal contents from the open abdominal site, with adhesion of foreign bodies such as hair, corn bedding, and intestinal contents to the mesh surface. Additionally, there was breakage observed on one side, accompanied by stained skin and severe contamination at the defective site and the surrounding area (Figure 3Bi). Group B rats had complete detachment of all external patches at the open abdominal site. The mesh remained intact without any breakage, while both the defective site and surrounding skin were dry (Figure 3Bii). In group C rats, there was evident invasion of a foreign substance at the open abdominal site. Notably, pus accumulation between the mesh and patch was observed (Figure 3Biii).

On POD 7, the rats in group A exhibited an evident mesh rupture and conspicuous contamination at the defect site (Figure 3Ci). Rats in group B showed partial detachment of the mesh, while the defect site appeared clean and moist, and displayed a tendency towards healing (Figure 3Cii). In group C, all patches were detached with partial mesh detachment observed along with slight pus contamination at the defect site (Figure 3Ciii). The wound healing rate results (Figure S1) also indicated that group B exhibited significantly superior wound healing compared to the other two groups.

After opening the abdominal cavity with a “U” shape incision on the left side of the abdomen,^11^ it was observed that all three groups exhibited adherence of the large omentum and intestinal tissues to the polypropylene mesh. Notably, group C displayed more pronounced intestinal obstruction compared to the other two groups, with a tendency towards bleeding (Figure 3D). The adhesion score results demonstrated no statistical difference between the three groups (*p* > 0.05). In addition, intestinal tube rupture was found in group A but not in the other two groups. Furthermore, after intestinal transection, the three groups exhibited varying degrees of fluid alterations in the intestinal contents. This observation implies that upon abdominal cavity exposure, the intestine is impacted which aligns with clinical observations.

### 3.3 X‐ray changes in the intestine

**FIGURE 4:**
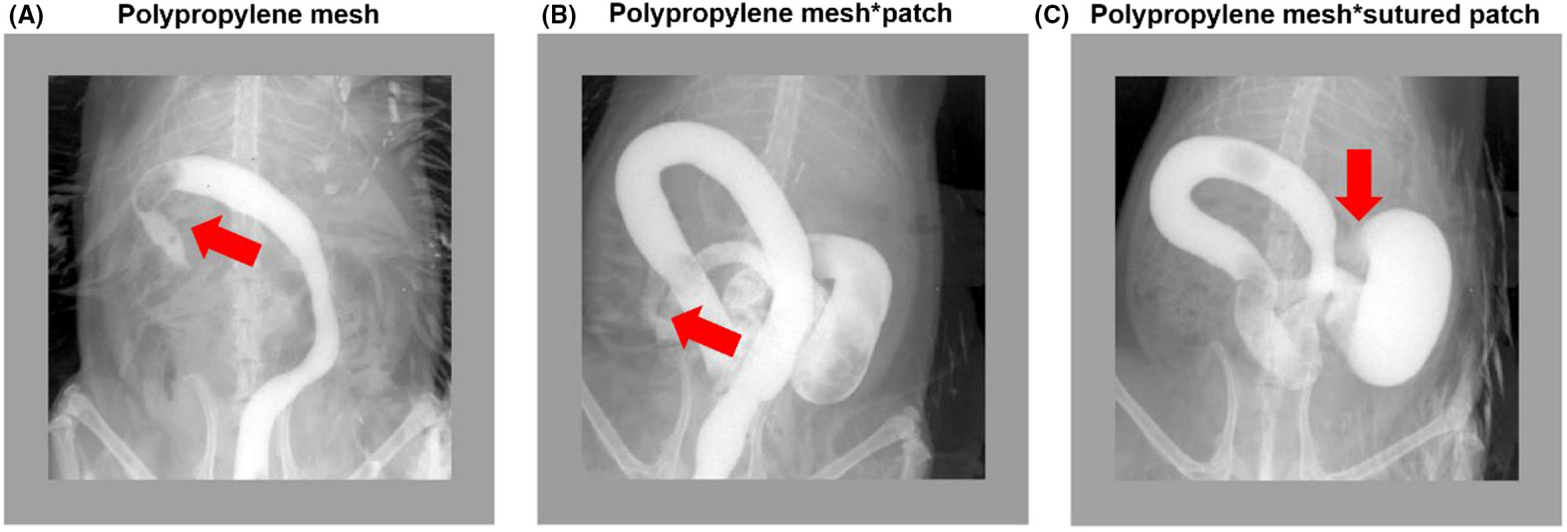
Abdominal X‐ray of rats on day 7 after OA. (A) The polypropylene mesh temporary abdominal closure group; (B) The polypropylene mesh*patch temporary abdominal closure group; (C) The polypropylene mesh*sutured patch temporary abdominal closure group; the red arrows show the intestinal obstruction.

Clinically, patients often develop abdominal adhesions and intestinal root obstruction or intestinal fistula after undergoing OA. We observed intestinal changes in rats following open abdominal cavity mapping by intestinal angiography. Intestinal angiography revealed different degrees of poor contrast patency and distal intestinal obstruction in all three groups, with more pronounced intestinal obstruction in rats in group C. This result was consistent with the general observations (Figure 4).

### 3.4 Histopathological changes

**FIGURE 5:**
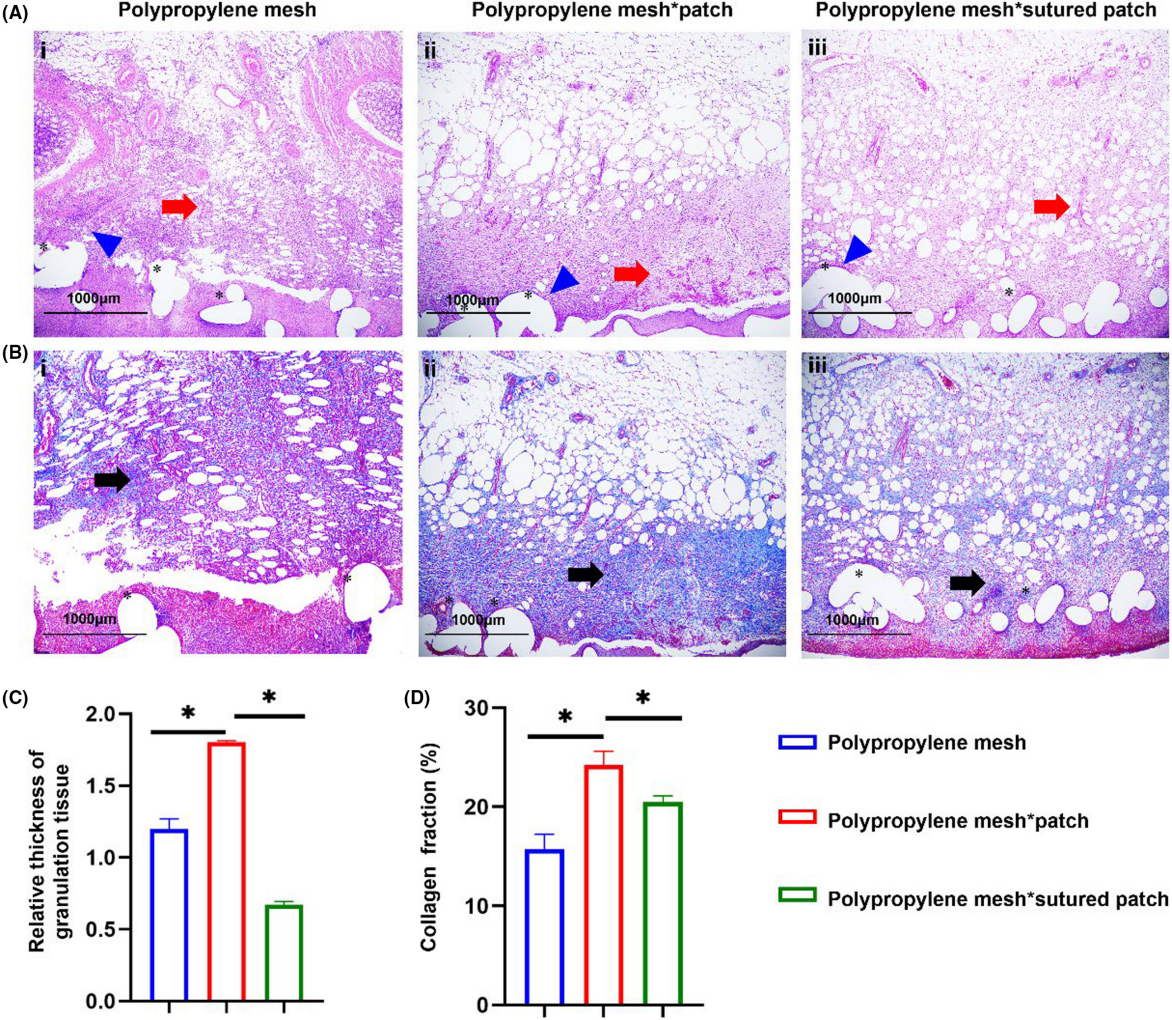
Histopathological features of rats on day 7 after OA. (A) HE staining of regenerative abdominal wall tissues (×4), blue triangles are inflammatory cell infiltration, * is the polypropylene mesh, and red arrows indicate neovascularization; (B) Masson staining of regenerative abdominal wall tissues (×4), black arrows indicate collagen fiber deposition. (C) The relative thickness of granulation following the three modeling methods, *p < 0.05. (D) The collagen fraction following the three modeling methods, *p < 0.05.

The postoperative wound healing of OA has consistently been a primary concern in clinical practice. The results of HE staining showed that the mesh was a partially absorbed fuzzy circular structure, surrounded by fibrous hyperplasia and infiltrated by inflammatory cells, mainly giant cells, lymphocytes, plasma cells, and eosinophils (Figure 5A). The rats in the three groups presented different degrees of granulation tissue and neovascularization in the abdominal wall, with group B revealing better granulation tissue and angiogenesis than the other two groups, and significantly different (*p* < 0.05) thickness of granulation tissue (Figure 5C). Masson staining revealed collagen deposition around the mesh in all three groups (Figure 5B). The collagen volume fraction in group B was significantly higher than that in the other two groups, revealing rapid collagen deposition (*p* < 0.05) (Figure 5D).

## 4 DISCUSSION

While it relieves intra‐abdominal hypertension, improves organ perfusion, and treats abdominal compartment syndrome (ACS) and severe abdominal infections, OA nonetheless poses many challenges for clinical management.^12^, ^13^ The complication rate after OA can be as high as 50%. Traumatic infections and intra‐abdominal abscess formation are particularly common in patients with multiple injuries, malnutrition and immunodeficiency.^14^, ^15^ This is the result of prolonged exposure of abdominal organs to air after abdominal opening and has been a limiting factor in the clinical promotion of open abdominal therapy.^16^, ^17^ Previous studies have shown that promoting open abdominal wound repair and restoring intra‐abdominal homeostasis as soon as possible can significantly reduce the incidence of complications and thus improve patient prognosis.^18^, ^19^ How to promote rapid wound repair has become an important direction in the current research on OA.^20^ However, there is still no reliable animal model to simulate the process of wound repair after OA.

In the present study, we adopted three methods to establish an animal model of OA. We found that the modeling method used in group B rats was significantly more efficient and reproducible than in the other two groups. The general condition of the rats in group B, including mental status, appetite, and recovery of urine and stool production, was also better than that in the other two groups. The results of HE staining also indicated that the wound healing in group B was better than in the other two groups, as evidenced by the thickness of new granulation tissue and neovascularization, and the collagen deposition around the mesh in group B was also higher than that in the other two groups. Thus, temporary closure of the abdomen with a patch (group B) is the best method for constructing an animal model of OA.

The method of constructing OA reported in previous literature has mainly been based on the temporary closure of the abdomen with simple polypropylene mesh, but this method has obvious limitations.^21^ Rats tend to tear the mesh, causing it to fall off and affecting the evaluation of experimental results. Therefore, we attached a medical wound waterproof patch (group B) and a sutured wound waterproof patch (group C) on top of polypropylene mesh to prevent mice from tearing the patches and preventing them from falling off. Foreign bodies such as hair and bedding tend to stick to the defect site, which makes it challenging to observe the general changes in the injury site and aggravates the inflammatory response in rats, even leading to infection and death. The disadvantages of the polypropylene mesh–sutured patch molding method (group C) include the presence of a gap between the mesh and the patch, which can easily lead to foreign body invasion, long‐term residuals and contamination of the abdominal cavity, and the mortality rate, which is still high. The sutured patch involves a more extended molding time and a more complicated method, and the potential harm to the body is unpredictable. The polypropylene mesh combined with the patch preparation method (group B) was an improvement on the simple polypropylene mesh construction method. This method constructed an effective model, and was also more convenient, simple, and reproducible. It also more realistically simulated the pathophysiological changes in clinical patients following OA, which is the ideal for establishing an OA model.

There are limitations in this study. Firstly, using rats as models introduces potential differences in physiological and anatomical characteristics compared to humans, which restricts the reliability of directly applying research results to humans. While we replicated the OA in healthy rats, we acknowledge the inherent limitation of not incorporating a comprehensive intra‐abdominal infection model. Furthermore, due to our limited housing conditions, the lack of control of movement after creating the rat model may have introduced external factors that affected how realistically our experimental results mimicked patient situations.^22^ To overcome these limitations, it is necessary to consider a broader range of research methods, including in vitro studies, ex vivo organ cultures, and clinical research, to gain a more comprehensive understanding of the biology and disease mechanisms involved in OA.

## 5 CONCLUSIONS

This study used a modified method consisting of a polypropylene mesh combined with a patch to establish an animal model of OA, to simulate the pathophysiological changes following OA. Our model provides an experimental basis for future research on the effects of OA and improved post‐operative treatment that is worthy of consideration.

## AUTHOR CONTRIBUTIONS

L.Y., W.X.W., and R.J.A. initiated and designed the research. L.Y., L.Z., H.J.J. developed animal model. C.K. and Q.G.W. detected and analyzed biochemical parameters. L.Y., L.S.C., and H.J.J analyzed and interpreted the data. L.Y., L.S.C., and W.X.W. wrote and revise the article. L.Y., W.X.W., and R.J.A. finally submitted paper.

## FUNDING INFORMATION

This study was funded by the National Natural Science Foundation of China (82270595), Jiangsu Provincial Medical Innovation Center (CXZX202217), and Postgraduate Research & Practice Innovation Program of Jiangsu Province (SJCX23_0092).

## ETHICS STATEMENT

The animal disposal and research protocols were approved by the Experimental Animal Ethics Committee of Nanjing Jinling Hospital, and the ethical approval document batch number was 2021DZDWLS‐001. All experiments were performed in compliance with Guide for the Care and Use of Laboratory Animals.

## AUTHOR STATEMENT

The authors stated that the manuscript including related data, figures, and tables has not been published previously.

## Supporting information


Data S1:

## Data Availability

The original data for this study can be accessed by the interested parties after contacting authors via the email address: jiananr@nju.edu.cn
