# Supplementary material for: Establishment and evaluation of an improved rat model of open abdomen
Source: Animal Model Exp Med. 2023 Dec 29;7(4):562–9. doi: 10.1002/ame2.12376 (PMC11369015; doi:10.1002/ame2.12376)
Supplement: Supplementary file 1 — Data S1: [file AME2-7-562-s001.docx]

**Establishment and Evaluation of an Improved Rat Model of Open Abdomen**

Ye Liu^1^, Sicheng Li^2^, Jinjian Huang^2^, Ze Li^2^, Kang Chen^2^, Guiwen Qu^1^, Xiuwen Wu^1,2^, Jianan Ren^1,2*^

1. School of Medicine, Southeast University, Research Institute of General Surgery, Jinling Hospital, Nanjing, 210009, China.

2. Research Institute of General Surgery, Jinling Hospital, Affiliated Hospital of Medical School, Nanjing University, Nanjing 210002, China.

Correspondence to:

Jianan Ren, e-mail: jiananr@nju.edu.cn

* Corresponding author.

**Table S1. General condition scoring chart of rats after OA**

| **Items** | **Score** |
| --- | --- |
| **Activity** |  |
| Completely inactive, unable to move | 1 |
| Activity significantly restricted | 2 |
| Mild restriction in activity | 3 |
| Normal activity level | 4 |
| **Appetite** |  |
| Completely not eating | 1 |
| Significantly reduced appetite | 2 |
| Mildly reduced appetite | 3 |
| Normal appetite | 4 |
| **Coat condition** |  |
| Disheveled coat, severe hair loss | 1 |
| Sparse coat, poor quality | 2 |
| Mildly sparse coat, average quality | 3 |
| Dense coat, good quality | 4 |
| **Mental state** |  |
| No response, unconscious | 1 |
| Slow response, decreased alertness | 2 |
| Mildly decreased alertness | 3 |
| Normal alertness | 4 |


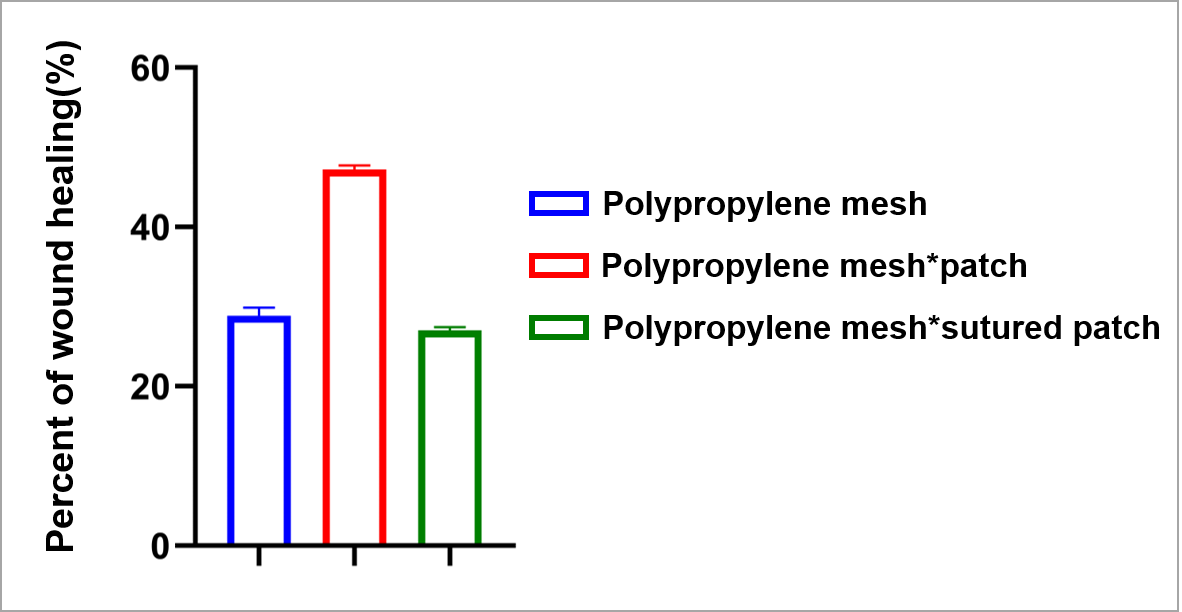


**Figure S1. The wound healing rate of the rats after OA.**
